# Association between menstrual-related disorders and sexually transmitted infections: A nationwide cross-sectional study in Japan

**DOI:** 10.1371/journal.pone.0351855

**Published:** 2026-06-16

**Authors:** Tatsuya Yoshihara, So Owada, Harumasa Arita, Akiko Nakagomi, Kota Tanaka, Yosuke Ono, Osamu Yoshino

**Affiliations:** 1 Department of Obstetrics and Gynecology, University of Yamanashi, Yamanashi, Japan; 2 Rohto Pharmaceutical Co., Ltd., Osaka, Japan; Faculty of Medicine Universitas Indonesia, INDONESIA

## Abstract

**Background:**

To investigate the association between menstrual-related disorders and sexually transmitted infections (STI) among young women in Japan, and to examine differences according to disorder type and hormonal therapy use.

**Methods:**

This cross-sectional study used the Japan Medical Data Center Claims Database and included women younger than 40 years who had at least one healthcare visit in 2023. Menstrual-related disorders were defined as endometriosis or dysmenorrhea based on ICD-10 codes. The prevalence of five STIs—gonorrhea, genital chlamydia infection, trichomoniasis, genital herpes, and other sexually transmitted conditions—was compared between women with and without menstrual-related disorders. Subgroup analyses were conducted for endometriosis, dysmenorrhea, and hormonal therapy (low-dose estrogen–progestin combinations or dienogest). Prevalence ratios (PR) and prevalence differences (PD) with 95% confidence intervals (CI) were estimated.

**Results:**

Among 3,440,929 women, 257,897 (7.5%) had menstrual-related disorders. All STI were substantially more prevalent in this group than in women without menstrual-related disorders, with PRs ranging from 4.31 to 5.29. Endometriosis showed the highest prevalence, particularly for genital chlamydia infection (4.98%; PR 7.44). Dysmenorrhea was also associated with consistently elevated STI prevalence. Among women with menstrual-related disorders, STI prevalence differed only slightly according to hormonal therapy use, with differences generally within one percentage point.

**Conclusion:**

Menstrual-related disorders were strongly associated with increased diagnosis of STI in Japanese young women. These findings highlight the importance of integrating STI screening and reproductive health education into routine gynecologic care for women with endometriosis or dysmenorrhea. The influence of healthcare-seeking behavior and diagnostic patterns should be considered when interpreting claims-based STI data.

## Introduction

Menstrual-related disorders, such as endometriosis and dysmenorrhea, are relatively common conditions among young women and represent significant health issues that can lead to chronic pelvic pain, reduced quality of life, and increased healthcare utilization [1–3]. These conditions often require hormonal therapies, such as low-dose estrogen–progestin combinations (LEP, corresponding to combined oral contraceptives reimbursed in Japan) or dienogest (DNG; 1–2 mg per day), a progestin-based agent, and are therefore associated with a relatively high frequency of gynecologic healthcare visits [4–6].

Sexually transmitted infections (STI) are highly prevalent among young women, and infections such as chlamydia and gonorrhea are major causes of future reproductive morbidity, including infertility and pelvic inflammatory disease [7,8]. However, few studies have examined the association between menstrual-related disorders and STI using large-scale claims data, and only a limited number have evaluated endometriosis and dysmenorrhea separately [7].

Furthermore, hormonal treatments for menstrual-related disorders, such as LEP and DNG, may influence sexual behavior, condom use, and patterns of healthcare access [6,9,10]. However, epidemiologic evidence regarding how these hormonal therapies affect the diagnosis of STI remains limited [7,11,12].

Based on these considerations, this study aimed to use large-scale Japanese claims data to: (1) examine the association between menstrual-related disorders and STI; (2) assess differences according to the type of menstrual-related disorders (endometriosis and dysmenorrhea); and (3) evaluate whether STI diagnosis patterns differ according to the use of hormonal therapy.

## Materials and methods

This cross-sectional study was conducted using the Japan Medical Data Center (JMDC) Claims Database, which contains health insurance claims primarily from employees of large-scale health insurance societies in Japan, and included data from January 1 to December 31, 2023. The JMDC database contains anonymized health insurance claims data, including information on healthcare utilization, ICD-10 diagnostic codes, and medication prescriptions. In accordance with the “Ethical Guidelines for Medical and Health Research Involving Human Subjects” in Japan, ethical approval and informed consent were not required because this study was a secondary analysis of a commercially available, completely anonymized database. The data are disconnected from any personal identifiers, and the study did not involve any intervention or contact with the participants.

The study population consisted of women younger than 40 years who had at least one healthcare visit during the study period. Menstrual-related disorders were defined as having at least one recorded ICD-10 diagnosis of endometriosis (N80) or dysmenorrhea (N944–N946), whereas women without these diagnoses were classified as the non–menstrual-related disorder group. We further classified menstrual-related disorders into two subgroups: endometriosis and dysmenorrhea. Regarding hormonal therapy, women were considered to have received hormonal treatment if they had at least one prescription for either LEP or DNG for their menstrual-related disorder during the study period. Women without any prescription records for LEP or DNG during the same period were classified as not receiving hormonal therapy. A formal washout period for prior hormonal therapy use before group classification was not applied.

The outcomes of interest were the following sexually transmitted infections: gonorrhea (A54), genital chlamydia infection (A56), trichomoniasis (A59), genital herpes (A60), and other sexually transmitted conditions (A63). For each infection, cases were considered positive if at least one diagnosis was recorded during 2023. For both menstrual-related disorders and STI, diagnostic codes indicating “suspected” conditions were excluded.

In the statistical analysis, we calculated the prevalence of sexually transmitted infections according to the presence or absence of menstrual-related disorders, the subgroups of endometriosis and dysmenorrhea, and the presence or absence of hormonal therapy. Between-group comparisons were performed using prevalence ratios (PR) and prevalence differences (PD). Ninety-five percent confidence intervals (CI) were estimated using the Wald method, and all analyses were conducted using JMP Pro version 18.2.0.

Because only aggregated tabulations were available for analysis, and individual-level records and detailed covariate information were not accessible, multivariable adjustment for potential confounders could not be performed. To provide additional context, supplementary age-stratified prevalence analyses were also conducted for the major STI outcomes using aggregated tabulations by age category. For each age stratum, prevalence, PR, PD, and their 95% CI were calculated using the same Wald-method framework as in the primary analyses.

## Results

A total of 3,440,929 women under 40 years of age had at least one healthcare visit during the study period. Among them, 257,897 were classified as having menstrual-related disorders based on a diagnosis of endometriosis (n = 96,863) or dysmenorrhea (n = 237,727), with overlap between the two conditions. 3,183,032 women had no menstrual-related disorder. Among those with menstrual-related disorders, 177,366 received a prescription for either LEP or DNG, whereas 80,531 did not receive hormonal therapy.

### 1. Association between menstrual-related disorders and sexually transmitted infections

Genital chlamydia infection (A56) was identified in 9,106 of 257,897 women with menstrual-related disorders (3.53%) and in 21,237 of 3,183,032 women without menstrual-related disorders (0.67%). The PR was 5.29 (95% CI, 5.17–5.42), and the PD was + 2.86 percentage points (95% CI, 2.79–2.94). Thus, chlamydia infection was diagnosed approximately 5.3 times more frequently in the menstrual-related disorder group than in the reference group.

Gonorrhea (A54) was identified in 2,225 of 257,897 women with menstrual-related disorders (0.86%) and in 5,426 of 3,183,032 women without menstrual-related disorders (0.17%). The PR) was 5.06 (95% CI, 4.82–5.32), and the PD was + 0.69 percentage points (95% CI, 0.66–0.73). Trichomoniasis (A59) was diagnosed in 1,675 of 257,897 women with menstrual-related disorders (0.65%) and in 4,330 of 3,183,032 women without menstrual-related disorders (0.14%), yielding a PR of 4.77 (95% CI, 4.51–5.05) and a PD of +0.51 percentage points (95% CI, + 0.48 - + 0.54). Genital herpes (A60) was observed in 2,086 of 257,897 women with menstrual-related disorders (0.81%) and in 5,829 of 3,183,032 women in the reference group (0.18%). The PR was 4.42 (95% CI, 4.20–4.64), and the PD was + 0.63 percentage points (95% CI, + 0.59 - + 0.66). Similarly, other sexually transmitted conditions (A63) were more frequent among women with menstrual-related disorders (0.34%) than in the reference group (0.08%), with a PR of 4.31 (95% CI, 3.99–4.65) and a PD of +0.26 percentage points (95% CI, + 0.24 - + 0.28) (Table 1).

**Table 1:** Prevalence of STI among women with and without menstrual-related disorders.

	Prevalence in the menstrual-related disorder group (%)	Prevalence in the non-menstrual-related disorder group (%)	PR	PR 95% CI	PD (%)	PD 95% CI
Gonorrhea	0.86	0.17	5.06	4.82–5.32	+0.69	+0.66– + 0.73
Genital chlamydia infection	3.53	0.67	5.29	5.17–5.42	+2.86	+2.79– + 2.94
Trichomoniasis	0.65	0.14	4.77	4.51–5.05	+0.51	−0.48– + 0.54
Genital herpes	0.81	0.18	4.42	4.20–4.64	+0.63	+0.59– + 0.66
Other sexually transmitted conditions	0.34	0.08	4.31	3.99–4.65	+0.26	+0.24– + 0.28

Comparison of the prevalence of STI between the menstrual-related disorder group and those without menstrual-related disorders.

Prevalence ratios (PR) and prevalence differences (PD) with 95% confidence intervals (CIs) were calculated using the Wald method.

CI, confidence interval; PD, prevalence difference; PR, prevalence ratio; STI, sexually transmitted infection.

Age-stratified prevalence analyses for gonorrhea, genital chlamydia infection, trichomoniasis, genital herpes, and other sexually transmitted conditions in women with and without menstrual-related disorders are presented in Supplementary Table 1 in S1 File, and the overall results were not materially changed after stratification by age.

### 2. Subgroup analyses of endometriosis and dysmenorrhea

In the endometriosis group, genital chlamydia infection was identified in 4,810 of 96,863 women (4.98%), which was markedly higher than in women without menstrual-related disorders (0.67%), with a PR of 7.44 (95% CI, 7.22–7.67) and a PD of +4.30 percentage points (95% CI, + 4.16 - + 4.44). Trichomoniasis was observed in 880 of 96,863 women (0.91%), corresponding to a PR of 6.68 relative to women without menstrual-related disorders. Gonorrhea, genital herpes, and other sexually transmitted conditions were also more common in the endometriosis group (1.09%, 0.77%, and 0.34%, respectively), with PRs generally ranging from approximately 3.6 to 5.5 (Table 2).

**Table 2:** Prevalence of STI in women with endometriosis compared with women without menstrual-related disorder group.

	Prevalence in the endometriosis group (%)	Prevalence in the non-menstrual-related disorder group (%)	PR	PR 95% CI	PD (%)	PD 95% CI
Gonorrhea	1.09	0.17	6.40	5.99–6.84	+0.92	+0.86– + 0.99
Genital chlamydia infection	4.98	0.67	7.44	7.22–7.67	+4.30	+4.16– + 4.44
Trichomoniasis	0.91	0.14	6.68	6.21–7.18	+0.77	+0.71– + 0.83
Genital herpes	0.77	0.18	4.18	3.88–4.51	+0.58	+0.53– + 0.64
Other sexually transmitted conditions	0.34	0.08	4.35	3.88–4.88	+0.26	+0.23– + 0.30

Comparison of the prevalence of STI between the endometriosis group and women without menstrual-related disorders.

Prevalence ratios (PR) and prevalence differences (PD) with 95% confidence intervals (CIs) were calculated using the Wald method.

CI, confidence interval; PD, prevalence difference; PR, prevalence ratio; STI, sexually transmitted infection.

In the dysmenorrhea group, genital chlamydia infection occurred in 7,609 of 237,727 women (3.20%), gonorrhea in 1,983 women (0.83%), trichomoniasis in 1,427 women (0.60%), genital herpes in 1,924 women (0.81%), and other sexually transmitted conditions in 797 women (0.34%). Each of these infections occurred at frequencies approximately 3–4 times higher than women without menstrual-related disorders (Table 3).

**Table 3:** Prevalence of STI in women with dysmenorrhea compared with women without menstrual-related disorder group.

	Prevalence in the dysmenorrhea group (%)	Prevalence in the non-menstrual-related disorder group (%)	PR	PR 95% CI	PD (%)	PD 95% CI
Gonorrhea	0.83	0.17	4.89	4.65–5.15	+0.66	+0.63– + 0.70
Genital chlamydia infection	3.20	0.67	4.80	4.67–4.92	+2.53	+2.46– + 2.60
Trichomoniasis	0.60	0.14	4.41	4.16–4.68	+0.46	+0.43– + 0.50
Genital herpes	0.81	0.18	4.42	4.20–4.65	+0.63	+0.59– + 0.66
Other sexually transmitted conditions	0.34	0.08	4.28	3.96–4.64	+0.26	+0.23– + 0.28

Comparison of the prevalence of STI between the dysmenorrhea group and women without menstrual-related disorders.

Prevalence ratios (PR) and prevalence differences (PD) with 95% confidence intervals (CIs) were calculated using the Wald method.

CI, confidence interval; PD, prevalence difference; PR, prevalence ratio; STI, sexually transmitted infection.

Overall, both endometriosis and dysmenorrhea were consistently associated with higher prevalences of multiple sexually transmitted infections compared with the reference group.

### 3. Comparison of sexually transmitted infections according to the use of hormonal therapy

Among women with menstrual-related disorders, we examined the association between hormonal therapy (LEP or DNG) and the diagnosis of sexually transmitted infections. Genital chlamydia infection was observed in 5,704 of 177,366 women receiving hormonal therapy (3.22%) and in 3,402 of 80,531 women not receiving hormonal therapy (4.22%). Although the prevalence was slightly higher in the non-hormonal therapy group, the difference was limited to approximately one percentage point. Gonorrhea was diagnosed in 0.85% of women receiving hormonal therapy and 0.90% of those not receiving it, while trichomoniasis occurred in 0.58% and 0.80%, respectively—differences that were minimal. The prevalences of genital herpes and other sexually transmitted conditions were also similar between the two groups, at around 0.8% and 0.3%, respectively, with no clear differences observed (Table 4).

**Table 4:** Prevalence of STI among women with menstrual-related disorders, comparing those with and without hormonal therapy.

	Prevalence in the hormonal therapy group (%)	Prevalence in the non-hormonal therapy group (%)	PR	PR 95% CI	PD (%)	PD 95% CI
Gonorrhea	0.85	0.90	0.94	0.86–1.02	−0.06	−0.13– + 0.02
Genital chlamydia infection	3.22	4.22	0.76	0.73–0.79	−1.01	−1.17– − 0.85
Trichomoniasis	0.58	0.80	0.72	0.66–0.80	−0.22	−0.29– − 0.15
Genital herpes	0.83	0.77	1.08	0.98–1.18	+0.06	−0.02– + 0.13
Other sexually transmitted conditions	0.35	0.30	1.16	1.00–1.34	+0.05	+0.00 −0.01

Comparison of the prevalence of STI between hormonal therapy users and non-users among women with menstrual-related disorders. Prevalence ratios (PR) and prevalence differences (PD) with 95% confidence intervals (CIs) were calculated using the Wald method.

CI, confidence interval; PD, prevalence difference; PR, prevalence ratio; STI, sexually transmitted infection.

Age-stratified prevalence analyses according to hormonal therapy use are presented in Supplementary Table 2 in S1 File, and the overall results were not materially changed after stratification by age.

## Discussion

In this study, we used large-scale Japanese claims data to examine the cross-sectional association between menstrual-related disorders and STI. Across all types of STI, women with menstrual-related disorders exhibited substantially higher prevalences approximately three to five folds compared with those without such disorders. Both endometriosis and dysmenorrhea were similarly and consistently associated with higher STI prevalence. In contrast, among women with menstrual-related disorders, differences in STI prevalence according to the use of hormonal therapy such as LEP or DNG were small, suggesting that hormonal treatment itself is unlikely to be a major determinant of STI occurrence.

Several factors may underlie the higher frequency of STI observed among women with menstrual-related disorders [6,7]. First, patients with dysmenorrhea or endometriosis tend to have more frequent gynecologic visits, which increases opportunities for STI screening and testing, leading to potential diagnostic bias [6,7,13]. In practice, menstrual-related disorders often prompt medical consultations due to pain, and regular follow-up is required for the management of hormonal therapy [2,5,14]. Consequently, even mild symptoms of STI—such as increased vaginal discharge or genital bleeding—are more likely to be detected through frequent contact with healthcare providers [7,8]. Second, menstrual-related disorders themselves may be associated with dyspareunia and inflammation, potentially affecting mucosal barriers and local immune responses [2,15,16]. Endometriosis, in particular, is known to be linked to peritoneal inflammation and sexual dysfunction, and some reports suggest that dyspareunia may influence contraceptive practices and sexual behavior [15]. These biological and behavioral factors may potentially contribute to the observed association [10,13]. However, these mechanisms remain speculative in the context of the present cross-sectional claims-based analysis. It is also possible that prior STIs, including pelvic inflammatory disease, contributed to pelvic pain, adhesions, or related symptoms that were subsequently diagnosed or coded as endometriosis or dysmenorrhea; therefore, reverse causality and diagnostic overlap may partly explain the observed association.

In the subgroup analyses, both the endometriosis and dysmenorrhea groups demonstrated higher prevalences of all sexually transmitted infections compared with women without menstrual-related disorders. The patterns of infection were generally similar between the two groups, with chlamydia, gonorrhea, trichomoniasis, genital herpes, and other sexually transmitted conditions occurring at levels approximately three to six times higher than in women without menstrual-related disorders. The relative risks were also largely comparable between the two groups. These findings suggest that, regardless of the specific type of menstrual-related disorder, shared underlying factors—such as more frequent healthcare visits, increased opportunities for testing, and behavioral changes related to sexual activity or pain—may contribute to the elevated rates of STI diagnosis.

Regarding the association between hormonal therapy for menstrual-related disorders and STI, the prevalence of chlamydia and trichomoniasis was slightly lower among hormonal therapy users; however, the differences were small and unlikely to be clinically meaningful. Although some hormonal therapy users may take the medication partly for contraceptive purposes—potentially leading to lower condom use—our findings did not provide clear evidence that hormonal therapy increases the likelihood of STI diagnosis [10,17,18]. However, women who do not use LEP and DNG may have fewer gynecologic visits, resulting in unequal opportunities for testing, and thus the possibility of undiagnosed or missed STI in this group cannot be excluded.

Supplementary age-stratified prevalence analyses were broadly consistent with the main findings. In particular, elevated prevalences of recorded STI diagnoses in women with menstrual-related disorders were observed across multiple age categories, whereas age-stratified differences according to hormonal therapy use were generally small, although these supplementary analyses remained unadjusted.

This study has several limitations. First, because of its cross-sectional design, causal inferences cannot be made, and the temporal relationship between menstrual-related disorders and STI diagnoses could not be determined. In addition, prior history or treatment of STI before the observation period could not be fully accounted for. Furthermore, some women with prior STI-related pelvic symptoms or pelvic inflammatory disease may have subsequently been diagnosed or coded as having endometriosis or dysmenorrhea, raising the possibility of reverse causality and diagnostic overlap. Second, due to the inherent constraints of claims data, important confounding factors—such as symptom severity, sexual behavior, contraceptive practices, number of sexual partners, frequency of sexual activity, and condom use—could not be adjusted for. These unmeasured behavioral factors may have confounded the observed associations. Third, detection bias related to differential healthcare-seeking behavior is an important concern. Women with menstrual-related disorders, particularly those receiving hormonal therapy, are likely to have more frequent contact with gynecologists and therefore more opportunities for STI testing and diagnosis than women without these disorders. Because the present database analysis could not adequately adjust for gynecologic visit frequency or healthcare-seeking intensity, part of the observed association may reflect differences in opportunities for detection rather than true differences in underlying STI prevalence. Moreover, because many STIs are asymptomatic, whether testing was performed likely had a substantial influence on diagnosis. Fourth, in Japan, oral contraceptives used solely for contraception are often provided as self-paid services and therefore are not captured in the claims database. As a result, some sexually active women using low-dose estrogen–progestin combinations for contraception but without menstrual-related disorders may have been misclassified into the non–menstrual-related disorder group, which may have diluted the contrast between groups and biased the prevalence ratios toward the null. Fifth, among women with menstrual-related disorders, the non-user group may have included some women who had previously used LEP or DNG and discontinued treatment before or during the study period, because a formal washout period was not applied. This potential misclassification may also have influenced the subgroup comparison according to hormonal therapy use.

This study is one of the first to comprehensively examine the association between menstrual-related disorders and recorded STI diagnoses among young women in Japan using large-scale claims data. Our findings suggest that young women with menstrual-related disorders may represent a clinically important group in whom sexual and reproductive health should be assessed carefully in routine gynecologic care. However, the observed associations should be interpreted cautiously because differential healthcare-seeking behavior, greater opportunities for STI testing, and unmeasured behavioral confounding may have influenced the results. Women with menstrual-related disorders often attend gynecologic clinics for pain-related symptoms or hormonal therapy follow-up, and STI-related symptoms such as abnormal discharge or genital bleeding may also increase opportunities for testing during these visits. In routine gynecologic practice, a comprehensive approach that includes reproductive health education and STI prevention counseling, in addition to symptom management, may therefore be beneficial for young women with endometriosis or dysmenorrhea.

## Supporting information

S1 File Supplementary tables. This file contains Supplementary Table 1 and Supplementary Table 2. Supplementary Table 1 presents age-stratified prevalence analyses of recorded STI diagnoses in women with menstrual-related disorders versus women without menstrual-related disorders. Supplementary Table 2 presents age-stratified prevalence analyses of recorded STI diagnoses according to hormonal therapy use among women with menstrual-related disorders.
(DOCX)
