# Supplementary material for: Association between menstrual-related disorders and sexually transmitted infections: A nationwide cross-sectional study in Japan
Source: PLoS One. 2026 Jun 16;21(6):e0351855. doi: 10.1371/journal.pone.0351855 (PMC13271507; doi:10.1371/journal.pone.0351855)
Supplement: S1 File — This file contains Supplementary Table 1 and Supplementary Table 2. Supplementary Table 1 presents age-stratified prevalence analyses of recorded STI diagnoses in women with menstrual-related disorders versus women without menstrual-related disorders. Supplementary Table 2 presents age-stratified prevalence analyses of recorded STI diagnoses according to hormonal therapy use among women with menstrual-related disorders. (DOCX) [file pone.0351855.s001.docx]

Supplementary Table 1. Age-stratified prevalence analyses of recorded STI diagnoses in women with menstrual-related disorders versus women without menstrual-related disorders

Age-specific prevalence, PR, PD, and 95% CI were calculated from aggregated tabulations using the Wald method.

CI, confidence interval; PD, prevalence difference; PR, prevalence ratio; STI, sexually transmitted infection.

| Outcome | Age group | Prevalence in exposed group (%) | Prevalence in reference group (%) | PR (95% CI) | PD, percentage points (95% CI) |
| --- | --- | --- | --- | --- | --- |
| Gonorrhea (A54) | <15 years | 0.02 | 0.00 | 9.57 (2.28–40.17) | 0.02 (-0.01 to 0.05) |
| Gonorrhea (A54) | 15–19 years | 0.74 | 0.17 | 4.41 (3.78–5.13) | 0.57 (0.47 to 0.67) |
| Gonorrhea (A54) | 20–24 years | 1.28 | 0.43 | 3.01 (2.77–3.27) | 0.86 (0.77 to 0.95) |
| Gonorrhea (A54) | 25–29 years | 0.93 | 0.35 | 2.68 (2.44–2.96) | 0.59 (0.51 to 0.66) |
| Gonorrhea (A54) | 30–34 years | 0.75 | 0.22 | 3.38 (2.99–3.82) | 0.53 (0.45 to 0.61) |
| Gonorrhea (A54) | 35–39 years | 0.56 | 0.15 | 3.77 (3.27–4.35) | 0.41 (0.34 to 0.48) |
| Genital chlamydia infection (A56) | <15 years | 0.13 | 0.00 | 49.14 (24.63–98.01) | 0.13 (0.05 to 0.20) |
| Genital chlamydia infection (A56) | 15–19 years | 2.13 | 0.46 | 4.61 (4.21–5.04) | 1.67 (1.50 to 1.83) |
| Genital chlamydia infection (A56) | 20–24 years | 4.60 | 1.41 | 3.26 (3.12–3.41) | 3.19 (3.02 to 3.35) |
| Genital chlamydia infection (A56) | 25–29 years | 4.17 | 1.44 | 2.90 (2.77–3.03) | 2.73 (2.57 to 2.89) |
| Genital chlamydia infection (A56) | 30–34 years | 3.51 | 1.13 | 3.12 (2.95–3.29) | 2.38 (2.22 to 2.55) |
| Genital chlamydia infection (A56) | 35–39 years | 2.82 | 0.72 | 3.91 (3.67–4.16) | 2.10 (1.95 to 2.25) |
| Trichomoniasis (A59) | <15 years | 0.02 | 0.00 | 17.87 (4.09–78.12) | 0.02 (-0.01 to 0.05) |
| Trichomoniasis (A59) | 15–19 years | 0.38 | 0.07 | 5.30 (4.26–6.60) | 0.31 (0.24 to 0.38) |
| Trichomoniasis (A59) | 20–24 years | 0.76 | 0.23 | 3.24 (2.91–3.61) | 0.52 (0.45 to 0.59) |
| Trichomoniasis (A59) | 25–29 years | 0.72 | 0.29 | 2.49 (2.23–2.78) | 0.43 (0.36 to 0.50) |
| Trichomoniasis (A59) | 30–34 years | 0.73 | 0.26 | 2.80 (2.48–3.16) | 0.47 (0.39 to 0.55) |
| Trichomoniasis (A59) | 35–39 years | 0.61 | 0.19 | 3.19 (2.80–3.64) | 0.42 (0.35 to 0.49) |
| Genital herpes (A60) | <15 years | 0.05 | 0.01 | 4.50 (1.66–12.20) | 0.04 (-0.01 to 0.08) |
| Genital herpes (A60) | 15–19 years | 0.46 | 0.11 | 4.21 (3.47–5.10) | 0.35 (0.28 to 0.43) |
| Genital herpes (A60) | 20–24 years | 0.93 | 0.32 | 2.90 (2.63–3.19) | 0.61 (0.53 to 0.68) |
| Genital herpes (A60) | 25–29 years | 0.97 | 0.35 | 2.75 (2.50–3.02) | 0.62 (0.54 to 0.70) |
| Genital herpes (A60) | 30–34 years | 0.86 | 0.32 | 2.66 (2.38–2.97) | 0.54 (0.45 to 0.62) |
| Genital herpes (A60) | 35–39 years | 0.74 | 0.27 | 2.68 (2.38–3.02) | 0.46 (0.38 to 0.54) |
| Other sexually transmitted conditions (A63) | <15 years | 0.01 | 0.01 | 1.84 (0.26–13.21) | 0.01 (-0.02 to 0.03) |
| Other sexually transmitted conditions (A63) | 15–19 years | 0.21 | 0.08 | 2.79 (2.13–3.67) | 0.14 (0.08 to 0.19) |
| Other sexually transmitted conditions (A63) | 20–24 years | 0.57 | 0.19 | 2.94 (2.59–3.32) | 0.37 (0.31 to 0.43) |
| Other sexually transmitted conditions (A63) | 25–29 years | 0.42 | 0.15 | 2.79 (2.41–3.23) | 0.27 (0.22 to 0.32) |
| Other sexually transmitted conditions (A63) | 30–34 years | 0.24 | 0.10 | 2.34 (1.90–2.88) | 0.13 (0.09 to 0.18) |
| Other sexually transmitted conditions (A63) | 35–39 years | 0.17 | 0.07 | 2.49 (1.95–3.19) | 0.10 (0.06 to 0.14) |

Supplementary Table 2. Age-stratified prevalence analyses of recorded STI diagnoses according to hormonal therapy use among women with menstrual-related disorders

Age-specific prevalence, PR, PD, and 95% CI were calculated from aggregated tabulations using the Wald method. Where one group had zero events, the PR confidence interval was not estimable.

CI, confidence interval; NE, not estimable; PD, prevalence difference; PR, prevalence ratio; STI, sexually transmitted infection.

| Outcome | Age group | Prevalence in hormonal therapy group (%) | Prevalence in non-therapy group (%) | PR (95% CI) | PD, percentage points (95% CI) |
| --- | --- | --- | --- | --- | --- |
| Gonorrhea (A54) | <15 years | 0.03 | 0.02 | 1.79 (0.11–28.57) | 0.01 (-0.06 to 0.09) |
| Gonorrhea (A54) | 15–19 years | 0.79 | 0.62 | 1.27 (0.94–1.71) | 0.17 (-0.03 to 0.37) |
| Gonorrhea (A54) | 20–24 years | 1.24 | 1.46 | 0.85 (0.72–1.00) | -0.22 (-0.45 to 0.01) |
| Gonorrhea (A54) | 25–29 years | 0.85 | 1.20 | 0.71 (0.59–0.84) | -0.35 (-0.54 to -0.16) |
| Gonorrhea (A54) | 30–34 years | 0.66 | 0.92 | 0.72 (0.58–0.88) | -0.26 (-0.43 to -0.09) |
| Gonorrhea (A54) | 35–39 years | 0.45 | 0.70 | 0.64 (0.51–0.82) | -0.25 (-0.39 to -0.11) |
| Genital chlamydia infection (A56) | <15 years | 0.20 | 0.09 | 2.14 (0.66–7.02) | 0.11 (-0.07 to 0.28) |
| Genital chlamydia infection (A56) | 15–19 years | 2.22 | 1.92 | 1.16 (0.98–1.38) | 0.31 (-0.04 to 0.65) |
| Genital chlamydia infection (A56) | 20–24 years | 4.36 | 5.59 | 0.78 (0.72–0.85) | -1.23 (-1.68 to -0.79) |
| Genital chlamydia infection (A56) | 25–29 years | 3.63 | 5.80 | 0.62 (0.58–0.68) | -2.18 (-2.59 to -1.77) |
| Genital chlamydia infection (A56) | 30–34 years | 2.72 | 4.85 | 0.56 (0.51–0.62) | -2.13 (-2.49 to -1.76) |
| Genital chlamydia infection (A56) | 35–39 years | 2.04 | 3.83 | 0.53 (0.48–0.59) | -1.79 (-2.10 to -1.48) |
| Trichomoniasis (A59) | <15 years | 0.00 | 0.04 | NE | -0.04 (-0.09 to 0.01) |
| Trichomoniasis (A59) | 15–19 years | 0.38 | 0.38 | 0.99 (0.66–1.47) | -0.00 (-0.16 to 0.15) |
| Trichomoniasis (A59) | 20–24 years | 0.69 | 1.06 | 0.64 (0.53–0.79) | -0.38 (-0.57 to -0.18) |
| Trichomoniasis (A59) | 25–29 years | 0.60 | 1.06 | 0.57 (0.47–0.69) | -0.46 (-0.64 to -0.28) |
| Trichomoniasis (A59) | 30–34 years | 0.61 | 0.94 | 0.64 (0.52–0.79) | -0.34 (-0.50 to -0.17) |
| Trichomoniasis (A59) | 35–39 years | 0.53 | 0.72 | 0.74 (0.59–0.93) | -0.19 (-0.33 to -0.05) |
| Genital herpes (A60) | <15 years | 0.10 | 0.02 | 5.36 (0.56–51.53) | 0.08 (-0.04 to 0.20) |
| Genital herpes (A60) | 15–19 years | 0.46 | 0.47 | 0.98 (0.68–1.40) | -0.01 (-0.18 to 0.16) |
| Genital herpes (A60) | 20–24 years | 0.88 | 1.11 | 0.79 (0.66–0.96) | -0.23 (-0.43 to -0.02) |
| Genital herpes (A60) | 25–29 years | 0.98 | 0.97 | 1.01 (0.84–1.22) | 0.01 (-0.17 to 0.19) |
| Genital herpes (A60) | 30–34 years | 0.88 | 0.83 | 1.06 (0.87–1.30) | 0.05 (-0.12 to 0.22) |
| Genital herpes (A60) | 35–39 years | 0.77 | 0.70 | 1.10 (0.89–1.36) | 0.07 (-0.08 to 0.22) |
| Other sexually transmitted conditions (A63) | <15 years | 0.03 | 0.00 | NE | 0.03 (-0.03 to 0.10) |
| Other sexually transmitted conditions (A63) | 15–19 years | 0.24 | 0.14 | 1.71 (0.93–3.14) | 0.10 (-0.00 to 0.20) |
| Other sexually transmitted conditions (A63) | 20–24 years | 0.55 | 0.64 | 0.85 (0.66–1.10) | -0.09 (-0.25 to 0.06) |
| Other sexually transmitted conditions (A63) | 25–29 years | 0.42 | 0.41 | 1.04 (0.78–1.38) | 0.02 (-0.10 to 0.13) |
| Other sexually transmitted conditions (A63) | 30–34 years | 0.21 | 0.29 | 0.72 (0.50–1.04) | -0.08 (-0.17 to 0.01) |
| Other sexually transmitted conditions (A63) | 35–39 years | 0.15 | 0.20 | 0.74 (0.48–1.15) | -0.05 (-0.13 to 0.03) |
